# Sleep apnea risk, subjective cognitive decline, and cognitive performance: Findings from the Boston Latino Aging Study (BLAST)

**DOI:** 10.1002/alz.093075

**Published:** 2025-01-03

**Authors:** Celina F. Pluim McDowell, Averi Giudicessi, Jairo E. Martinez, Alex L Badillo‐Cabrera, Nikole A Bonillas Félix, Lusiana Martinez, Diana Munera, Clara Vila‐Castelar, Nadine A Schwab, Liliana A Ramirez Gomez, Marta Gonzalez Catalan, Jeanne F. Duffy, Alice Cronin‐Golomb, Yakeel T. Quiroz

**Affiliations:** ^1^ Massachusetts General Hospital, Harvard Medical School, Boston, MA USA; ^2^ Brigham and Women’s Hospital, Harvard Medical School, Boston, MA USA; ^3^ Boston University, Boston, MA USA

## Abstract

**Background:**

Sleep apnea is associated with risk of objectively‐measured cognitive decline and dementia, as well as with subjective cognitive decline (SCD), itself a risk factor for cognitive decline and dementia. This relationship is understudied in ethnoracially diverse groups, however, including Latinos. This study examined associations among self‐reported sleep apnea risk, SCD, and cognitive performance in community‐dwelling older Latino adults.

**Method:**

Fifty‐six participants (39 men, 17 women) from the Boston Latino Aging Study (BLAST) were included (M_age_ = 66.5 years [SD = 8.6]; M_education_ 10.5 years [SD = 5.2]. Participants were administered the Preclinical Alzheimer’s Cognitive Composite‐5 (PACC5) (z‐scores), the Berlin Questionnaire to assess sleep apnea risk, in which positive scores in at least 2 categories (snoring and cessation of breathing; excessive daytime sleepiness; BMI and hypertension) indicates high risk, and the Cognitive Function Instrument (CFI) to assess SCD, in which higher scores indicate greater SCD. The Mini‐Mental State Examination (MMSE) measured global cognition. Pearson or point‐biserial correlations assessed associations among demographic factors (e.g., age, education), sleep apnea risk, SCD, and cognitive performance. T‐tests examined demographic and cognitive differences between those at high/low risk of sleep apnea.

**Result:**

On average, participants had intact global cognition (MMSE; M = 26.6 [3.3], range 16‐30) and mild SCD (CFI; M = 4.3 [3.8]). Thirty participants were classified as high risk for sleep apnea. Older age (*r* = ‐.35, *p* = .037) and fewer years of education (*r* = .68, *p*<.001) correlated with worse PACC5 performance. There was a trend for an association between high sleep apnea risk and worse PACC5 performance (*r =* ‐.32, *p* = .058). No other associations among demographics, sleep apnea risk, and cognition were significant (*p*’s>.16). Relative to those at low risk, those at high risk for sleep apnea had greater SCD (*p* = .018) and worse global cognitive performance (*p =* .019).

**Conclusion:**

These findings suggest that self‐reported sleep apnea risk may signal risk for cognitive decline in older Latinos. Screening for sleep apnea using a questionnaire may help identify those experiencing early cognitive decline or at risk of future decline. More work with larger sample sizes is needed to confirm these associations; BLAST data collection is ongoing.

